# Authors' Response to Peer Reviews of “Mask Use to Curtail Influenza in a Post–COVID-19 World: Modeling Study”

**DOI:** 10.2196/37146

**Published:** 2022-05-27

**Authors:** Henri Froese, Angel G A Prempeh

**Affiliations:** 1 Goethe-University Frankfurt am Main Frankfurt Germany; 2 Saginaw Valley State University Saginaw, MI United States

**Keywords:** mask, protection, COVID-19, influenza, transmission, intervention, infectious disease, respiratory, simulation, model, prevalence, efficacy

*This is a peer-review report submitted for the paper “Mask Use to Curtail Influenza in a Post–COVID-19 World: Modeling Study.”*

## Round 1 Review

### Anonymous [1]

#### Major Comments

1. An additional introductory paragraph on the susceptible-exposed-infected-recovered (SEIR) model would strengthen the manuscript [2] and open it up to a wider audience, as this topic is of interest to many.

Response: To address this, an introductory paragraph about the basic underworking of SEIR models has been added to the Methods section under the SEIR Model and Parameters subsection.

2. An additional 1 to 3 paragraphs in the Discussion are needed, comparing this study to similar studies.

Response: At the time of this write up, there have not been any similar studies, at least with comparable parameters with which we could compare.

3. I suggest the authors use color-blind–friendly colors for the figures.

Response: The colors in the figures are yellow and green combinations as well as red and blue combinations to be color-blind–friendly.

#### Minor Comments

4. This statement needs rewording: “vaccines of course only have to be administered once while face masks need to be worn continuously.” I suggest separating this away from the rest of the sentence and making it a cleaner statement.

Response: The statement has been separated and has now been changed to “Nevertheless, vaccines only have to be administered once per year while face masks would need to be worn continuously.”

5. The last sentence of the Discussion is a run-on sentence. Please fix.

Response: The sentence has been broken down into different sentences to address the suggestion.

### Reviewer AL [3]

1. The final sentence of the Abstract needs to be completed or reworked to explain “other practical aspects.”

Response: The statement has been deleted. Other practical masks have been explained.

2. I’m assuming that this is all focused on solely the United States since it is using Centers for Disease Control and Prevention (CDC) data. However, noting that this is US-centric and giving a brief description of how the CDC acquires this data will help the reader understand the data set, especially with many of the CDC data sets being underrepresentative of actual case rates because they are highly dependent on medical reports. In the case of the flu, how many people get the flu but never report to the CDC or see a doctor to get treatment because symptoms are mild?

Response: A brief discussion of how the CDC obtains flu data through the National Notifiable Diseases Surveillance System has been added to the SEIR parameters subsection. We also address the issue of underreporting. The CDC acknowledges that since flu cases are heavily based on reports from hospitals, it is prone to underreporting as the reviewer suggested. This is why it undergoes further analysis to correct for this phenomenon. Details of the arithmetic and statistical manipulations are addressed in the revision paper.

3. A creation of a table of or explicitly stating the variables and values used in the model is important for understanding. Especially when it comes to the calculated variables like B(t). Is that the same for each of those curves or is it changing with the different curves? If so, how much does it vary?

Response: Since the values are temporal, we have referenced the source of the data set. A table of the values and model has been added to the SEIR parameters subsection of the paper; the calculated variables like B(t) remain constant.

#### Minor Comments

4. How much does the virulence of the flu strains for that year versus the efficacy of the vaccine that year affect the data you are working with? Are there years that you think the masks would have helped substantially more than other years because the vaccine efficacy was lower than expected?

Response: For concerns of overcomplexity, we did not consider variance in infection of different flu strains in this paper. Data used in all out work was extrapolated from the CDC combined influenza data.

On masks and vaccines, the primary objective was to highlight how masks could have helped. Surely, in times when vaccine efficacy was lower, masks could have been a reasonable option, but with the rate of flu transmission, it would also depend on the percentage of mask wearers.

5. What is the typical mask efficacy for respiratory viruses? How does this “real-world” efficacy rate compare to the efficacy rates that you are using in your model?

Response: We were very considerate of real-world efficacy rates, especially considering the political and social pushback against mask mandates. As such, we used a generous mask efficacy rate to account for shortcomings and other real-life issues that may arise outside of a controlled environment.

## Abbreviations

CDC: Centers for Disease Control and Prevention
SEIR: susceptible-exposed-infected-recovered
